# Adolescent healthcare: I’m Lovin’ it

**DOI:** 10.4102/sajhivmed.v21i1.1147

**Published:** 2020-10-21

**Authors:** Carey Pike, Connie Celum, Linda-Gail Bekker

**Affiliations:** 1 Desmond Tutu Health Foundation, Faculty of Health Sciences, University of Cape Town, Cape Town, South Africa; 2 Department of Global Health, University of Washington, Seattle, United States of America; 3 Department of Medicine, University of Washington, Seattle, United States of America; 4 Department of Epidemiology, University of Washington, Seattle, United States of America

The field of adolescent health is motivated by the potential to deliver healthcare interventions that could have long-term prevention and health promotion benefits.^1^ In South Africa (SA), adolescents (aged 10–24 years) are the largest growing demographic and yet show sub-optimal health outcomes, particularly those related to sexual and reproductive health (SRH).^2^ Interventions to facilitate risk awareness and access to testing and screening programmes reduce sexually transmitted infection (STI) rates and avoid unintended pregnancies, yet adolescents continue to face real and perceived barriers whilst accessing services.^3^

Modern adolescents differ from previous generations in mindset and behaviour. Driven by technological advances and urbanisation, the iGeneration (born between mid-1990s and mid-2000s) has heightened expectations of speed, functionality and choice. Whilst these characteristics are known and capitalised on by commercial enterprises, they are underutilised by healthcare platforms. Understanding the adolescent perspective can illuminate ways healthcare delivery can be adapted to promote adolescent access.

Here we compare the delivery of adolescent SRH services to that of fast-food outlets, which constitute a well-utilised service with competitive global revenues and a large adolescent clientele. We consider eight potential reasons fast food is popular amongst adolescents and how these concepts could be extended to optimise healthcare delivery and increase uptake.

1. *Are we there yet?* Long waiting times at healthcare facilities, influenced by patient–nurse ratios, type of service and time of arrival, are a barrier to healthcare services.^4^ While the average waiting time in a SA clinic is 116 min, the average fast-food client would only wait 255 seconds (just over 4 min).^5^ Although not directly comparable, the efforts that fast-food outlets undertake to promote fast service are relevant. The fast-food systems are signposted and easy to navigate, with available services clearly displayed to facilitate informed decision-making immediately from the queue. Clear healthcare information strategies, including appropriate infographics, inside and outside the clinics could similarly streamline visits.^3^ The fast-food production is optimised by efficient, automated machinery; the healthcare analogy is the point-of-care diagnostic systems and the same-day treatment, which would increase clinic efficiency, treatment initiation and retention. An example of a popular consumer-friendly healthcare model that provides convenient, rapid STI/HIV services with improved patient outcomes is the Dean Street Express clinic in London.^6,7^ A similar model could be adapted for adolescents.
2. *Location, location and location.* A convenient geospatial location is a high priority for fast-food outlets who prioritise foot traffic and proximity to transport routes, which are positively associated with increased uptake.^8^ Decentralisation of healthcare services through mobile clinics and school health programmes that provide screening services, medication collection and basic treatment with onward referrals could increase spatial access and decongest facilities. Off-site centres that provide quick, easy access may be particularly desirable to adolescents.^9^ The fast-food industry has further found that home delivery services are the easiest way to decongest their outlets. Medication delivery for chronic care patients could provide the health equivalent to UberEats – a platform that adolescents are very familiar with. Convenient operating hours also allow fast-food outlets to accommodate a broad range of schedules. Public health facilities that cater to the same broad population are sometimes inconveniently located and often operate solely during working hours, requiring patients to miss work or school. Extending operating hours into the early morning, late evening, and weekends to accommodate patients could promote uptake.
3. *Tell me what’s out there.* Each year, the average American child will view over 250 McDonalds’ advertisements exclusively tailored for their age group.^8^ Marketing for fast-food products is pervasive, which normalise the product being advertised and provide continuous reminders to access them. Advertising campaigns for HIV products that make use of multiple media platforms could accelerate public knowledge and normalise use.^9^ Different adverts are needed to target diverse population groups differentiated by age. To target health promotion media for youth, social media adverts and influencers (users who have an established audience and are able to promote products because of their perceived authenticity) could raise awareness of health interventions and normalise their use.^10^
4. *Life Tastes Good*: This was originally a 2001 campaign slogan for Coca Cola. This phrase captures the fast-food industry’s main message: this tastes good and by eating it your life will be good too. The message is gain framed, with an emphasis on the reward. Biomedical products for HIV such as antiretroviral therapy, oral pre-exposure prophylaxis (PrEP), voluntary male medical circumcision (MMC) and condoms have many positive benefits, yet are often offered within a negative framework that focuses on risk aversion and the negative consequences of not using the product. HIV, STI and pregnancy prevention might benefit from gain-frame messages such as the ‘Do what you want. Do it with love, respect, and condoms’ slogan employed by youth-led Youth Against AIDS organisation.^11,12^
5. *Fast food is cheap*, as are public healthcare services in SA. However, indirect costs such as transport to the facility and opportunity costs for missing work or school constitute real barriers to access. These costs could be circumvented through decentralised healthcare services that operate at flexible hours.
6. *Fast-food outlets are places to socialise.* Outlets benefit when customers are happy to be seen there. Building positive social associations with attendance is a way in which clinics could attract healthy individuals and patients into the space. This can be achieved by making the healthcare spaces attractive and adolescent friendly, normalising visits and offering linkage to in-person or virtual peer support linkages. Health promotion is supported by regular health visits, which are more accessible when quick, delivered by friendly, non-judgemental staff, and provided an overall positive experience.^3^
7. *Providing choice*: Fast-food outlets offer menus that can be tailored to preferences and specific dietary requirements. The HIV response is well-positioned to offer choice, particularly around prevention options. Choices can be tailored to provide an ‘adolescent menu’ of oral PrEP, condoms and MMC or a menu for sexually and gender diverse persons, including condoms, lubricant oral PrEP, MMC and, when they become available, effective rectal microbicides. Choices can change as your risk or preference changes. Promoting choice supports long-term engagement in the service.
8. *Keep up.* The fast-food industry is constantly evolving to meet customers’ dynamic demands. As the proportion of the population identifying as vegetarian has increased, the fast-food outlets have adapted to offer vegetarian meals. Responsiveness to customer feedback and popular trends is prioritised, with a focus on user-driven product development. Conversely, within healthcare a pathogenic focus can overshadow user-demand considerations. Although biomedical and behavioural interventions take years to move from concept to market, reducing their ability to be responsive, healthcare promotion and delivery can adapt to meet users’ needs and preferences.

Among adolescents, healthcare is one of many competing priorities. This makes it easy to put off clinic visits and regular health checks. As sub-Saharan Africa continues to develop and secure access to low-cost, fast services, the modern African adolescent may not only demand but start to expect healthcare delivery systems that fit their faster environment.
